# Health-related factors leading to disabilities in Korea: Survival analysis

**DOI:** 10.3389/fpubh.2022.1048044

**Published:** 2022-12-22

**Authors:** TaeEung Kim, So-Youn Park, In-Hwan Oh

**Affiliations:** ^1^Department of Preventive Medicine, School of Medicine, Kyung Hee University, Seoul, South Korea; ^2^Department of Medical Education and Humanities, School of Medicine, Kyung Hee University, Seoul, South Korea

**Keywords:** health-related factors of disabilities, comorbidity, chronic diseases, longitudinal study, Korea

## Abstract

The purpose of this study is to analyze (a) population and socioeconomic factors affecting disability, excluding the occurrence of disability due to accidents and congenital diseases, and (b) health-related behavioral factors and factors that can prevent and reduce the cause of disability due to disease in Korea. This study was a longitudinal research. Data were obtained from The 2018 Korean Health Panel (KHP) is a survey jointly conducted by the Korea Institute of Health and Social Affairs and the National Health Insurance Service. A total of 7, 372 (*M*age = 52.14, SD = 21.39; Male = 47.52%) were analyzed in this study. People with Higher education attainments and more income levels were associated with lower hazard of developing new disabilities (all *p* < 0.05). In this study, the health factors that could be related to the occurrence of new disabilities were smoking, alcohol consumption, physical activity, and stress (all *p* < 0.0001). However, physical activity was negatively associated with the risk of developing a disability at all follow-ups (*p* < 0.05). Higher scores on the number of chronic diseases (valid scores = 0, 1, 2, 3, or more) represented a greater level of newly developing disability present at all follow-ups (all *p* < 0.0001). This longitudinal study confirmed the relationship between health-related factors and specific chronic diseases. Its findings can be used as a crucial foundation for establishing healthcare policies and services that can lower and prevent disability by preventing and reducing specific negative health behaviors and unhealthy behavioral factors, and alleviating chronic diseases in Korea.

## 1. Introduction

As per a recent report by the World Health Organization (WHO), more than 1 billion people live with some form of disability, of which nearly 200 million face significant physical and mental difficulties (1). Almost everyone is likely to experience some form of disability (temporary or permanent) at some point in their lives; however, it has been found that the COVID-19 pandemic has disproportionately affected people with disabilities (1). The number of people with disabilities is increasing rapidly owing to demographic trends and an increase in chronic health conditions (e.g., diabetes, cardiovascular disease, cancer, and mental health disorders), among other causes (2). The global direct cost of all disabilities among individuals was between 11 and 69% of the total income, and the cost accounted for by the governments of the Organization for Economic Cooperation and Development (OECD) countries was ~10% of public social spending (2).

According to the Korea Disabled Persons Survey, as of May 2020, ~260 million people with disabilities (5.1% of the total population) are registered. As of 2020, the number of people with disabilities had increased by about 420,000, showing a steady increase compared to 2017 (3). According to an analysis of the socioeconomic costs of major diseases for health insurance policy establishment in 2017 (4), the socioeconomic cost of disability is significant to a societal set-up, and in Korea, it was observed that it substantially burdened the country's economy (4). In terms of the socioeconomic costs of mental and behavioral disorders in Korea, direct costs amounted to KRW 7.557 trillion (~$6.685 billion) and indirect costs to KRW 3.7705 trillion (about $3.335 billion) (4). Medical expenses and productivity loss accounted for a large portion of socioeconomic costs due to disability.

Disability types are influenced by trends in the individual's various health conditions and in environmental and other factors, such as road traffic accidents, natural disasters, conflict, diet, and substance abuse (5). Results from the World Health Survey show that the prevalence of disability in lower-income countries is higher than that in high-income countries. People in the poorest quintiles of wealth, women, and older adults also had a higher prevalence of disability (1). People who are unemployed and those with low incomes or those poorly educated are at a higher risk of disability (1, 2).

Occupational accidents, traffic accidents, and diseases have been commonly reported as the causes of disability. Diseases, such as cancer, heart attack, tuberculosis, pneumonia, diabetes, and cerebrovascular disease, are the most common causes of these disorders (5). Musculoskeletal disorders are the leading cause of disability, including arthritis, back pain, spinal/joint disease, and fibromyalgia (5). Furthermore, obesogenic lifestyle choices and personal behaviors (e.g., lack of exercise, high-calorie intake, sedentary lifestyle, smoking, alcohol consumption, stress, and psychological instability) are leading contributors to newly developing disabilities (5).

Previous studies that examined factors related to the development of various disabilities, except for war, terrorism, and traffic accidents, include original studies (6–52) and meta-analyses and review research (53–57). Most studies have investigated the socioeconomic characteristics [e.g., income level, (8, 9, 14, 17, 20) and education level (8, 14, 17, 18, 20)] of the population and the development of disability with respect to specific age and status [e.g., children, (10, 20) young and middle-aged adults, (14, 16, 19, 26, 42, 48, 50) veterans (35), and seniors and the older adults (6–8, 11, 17, 18, 22, 25, 28, 31, 32, 34, 39)]. Furthermore, many studies have identified that certain chronic diseases like hypertension, hyperlipidemia, joint, kidney and diseases [e.g., hypertension, (8, 19, 22, 30, 41, 43) hyperlipidemia (19), diabetes, (8, 30, 35, 36, 40, 41, 51, 55, 57) cerebrovascular disease (18, 30, 51), respiratory and lung disease (15, 30, 43, 45, 50, 57), cardiovascular disease (11–13, 30, 32, 45, 57), heart disease (35, 41, 46, 51), hip joint disease (30, 58), tuberculosis (54), kidney disease (44), HIV (14), cancer (30, 41, 57), and BMI/obesity (9, 19, 21, 22, 26, 27, 29, 32, 36, 39, 41, 45), etc.] or health-related factors, behaviors and choices [e.g., level of physical activity (6–9, 16, 27, 28, 31–34, 38, 42, 44, 56), smoking (9, 19, 32, 48, 49, 56), alcohol consumption (9, 32, 56), stress, anxiety, depression and dementia (8, 18, 30–32, 40, 45–47, 52)], are associated with a specific disabilities [e.g., physical disability (11, 16, 22, 25–31, 34, 35, 38–41, 49, 55, 59, 60), visual impairment (8, 10), hearing impairment (8), intellectual disability (41), developmental disability (41), mental disorder (8, 12, 13, 41, 56), respiratory disorder (15)].

Many studies have been conducted on various chronic diseases (disorders) and health-related behaviors; however, only a handful have analyzed the factors leading to the development of new disabilities. Thus, only a little is known about the risk of disability associated with health-related factors and chronic diseases in the development of new disabilities. Although several reviews (53–57, 61) and studies on the factors of the development of disability (6–52, 62) have summarized the evidence of a relationship between specific chronic disorders, health-related behaviors, and disability, no analysis thus far has estimated this risk in a longitudinal setting. Accurate estimation of the risk of disability associated with these factors is pivotal to understanding the health requirements that help with the prevention of disability onset among the concerned population.

This study analyzes (1) population and socioeconomic factors affecting disability, excluding the occurrence of disability due to accidents and congenital diseases, and (2) health-related behavioral factors and factors that can prevent and reduce the cause of disability due to disease. The specific research questions were as follows.

a. Health-related factors that can precipitate obesity, including smoking, alcohol consumption, lack of physical activity, and stress, are related to newly developed disabilities.

b. Chronic diseases (e.g., hypertension, diabetes, hyperlipidemia, arthropathy, tuberculosis, ischemic heart disease, and cerebrovascular disease) are associated with the new occurrence of disability.

## 2. Methods

### 2.1. Data source

The Korean Health Panel (KHP) (63) is a survey jointly conducted by the Korea Institute of Health and Social Affairs and the National Health Insurance Service and is utilized as basic data for establishing healthcare policies and health insurance policies by identifying changes in healthcare costs and medical expenditures. The first survey began in 2008, and as of January 2021, currents data was released to the 13th in 2018. The number of households to which the first survey (first half of the 2008 survey) was administered was 7,866 and the number of household members is 24,616. In the 13th survey in 2018, the number of households was 5,838, including 4,232 (equivalent to the first sample) and 1,606 (equivalent to the second sample) households. The total number of household member was 17,008. The rate of maintenance of the original households in 2018 was 53.8% in the first sample and 72.3% in the second sample.

The KHP survey method is based on the computer-assisted personal interviewing (CAPI) method, in which researchers visit households to be surveyed and respond to the questions using laptops (63). For the composition and training of the survey team, a surveyor in charge of panel households in each region was assigned, and training was conducted on the survey contents and guidelines. A health household account book for 1 year of medical use was prepared by panel households, and receipts and year-end settlement data were collected. A CAPI-based survey was conducted by visiting panel households, and health account books and medical receipts collected from panel households were checked. As a result of the household and household member survey responses, overall cleaning was performed on the medical use history and receipt data collected by households. Annual data has been constructed based on the survey responses and medical usage details entered by panel households and surveyors.

The Korean medical panel is divided into two broad groups: the contents of the survey for households and those of the personal survey for individual household members. For households, we investigated the household's socioeconomic characteristics, income and expenditure status, contents of medical service use, cost of purchasing general medicines, status of private medical insurance, and surveyed individual household members on chronic disease management, health level, health lifestyle, and medical accessibility.

The relevant institutional review board approved this study. All study participants agreed to participate in information provision (written or spoken) and to disclose information provision (written or spoken) data.

### 2.2. Study participants

The disability information presented in the Korea Health Panel Survey data (63) is divided into four categories, as shown in Table 1. During the study period, each year the participants were asked whether they had a disability, which reflected their existing disability status. The first category comprises the attributes of a disabled individual, who has been diagnosed with a disability and is registered with the Ministry of Health and Welfare as a disabled person (64). The second category is of no disability, and the third category highlights the attributes of a disabled person who has been diagnosed with a disability but has not been registered as a disabled person with the Ministry of Health and Welfare. The last category incorporates the status of a disabled individual who is not registered as disabled, and not judged by the Ministry of Health and Welfare as one or those awaiting a decision regarding the disability (64). In 2008 and 2009, participants were not asked whether they had a disability, and Table 1 shows the detailed status of disability registration in the Korea Health Panel (2008–2018).

**Table 1:** Korea health panel registration of persons with disabilities (63).

**Year**	**2008**	**2009**	**2010**	**2011**	**2012**	**2013**	**2014**	**2015**	**2016**	**2017**	**2018**
(1) Determination of disability + registration	0	0	*N =* 913 (5.10%)	*N =* 944 (5.54%)	*N =* 880 (5.54%)	*N =* 839 (5.56%)	*N =* 1,073 (5.58%)	*N =* 1,039 (5.73%)	*N =* 1,020 (5.85%)	*N =* 1,039 (6.05%)	*N =* 1,045 (6.14%)
(2) No disability	0	0	*N =* 16,972 (94.90%)	*N =* 16,046 (94.19%)	*N =* 14,949 (94.18%)	*N =* 13,957 (94.06%)	*N =* 18,091 (94.13%)	*N =* 17,057 (94.08%)	*N =* 16,373 (93.97%)	*N =* 16,111 (93.76%)	*N =* 15,929 (93.80%)
(3) Determination of disability + non-registration	0	0	0	*N =* 8 (0.05%)	*N =* 11 (0.07%)	*N =* 11 (0.07%)	*N =* 14 (0.07%)	*N =* 14 (0.08%)	*N =* 10 (0.05%)	*N =* 10 (0.06%)	*N =* 8 (0.05%)
(4) Disabled, not determined + not registered	0	0	0	*N =* 37 (0.22%)	*N =* 32 (0.02%)	*N =* 32 (0.22%)	*N =* 41 (0.21%)	*N =* 20 (0.11%)	*N =* 24 (0.13%)	*N =* 24 (0.14%)	*N =* 26 (0.15%)
Total	21,283	19,153	17,885	17,035	15,872	14,839	19,219	18,130	17,424	17,184	17,008

Source: The Korea Health Panel Survey (KHPS), 2008–2018; N, Number of participants.

This study included the following 15 types of disabilities designated by the Korean Ministry of Health and Welfare (64) including physical disabilities, brain lesion disorders, visual impairments, hearing impairments, speech disorders, intellectual disability, developmental disorders, mental disorders, kidney disorders, heart disorders, respiratory disorders, liver disorders, facial disorders, elongated and urinary tract disorders, and epilepsy disorders. For this study, along with disabled individuals, identified by the Korean government, we also included persons with disabilities who were not officially diagnosed and confirmed by the government. This is because it takes ~2 years in Korea to collect data regarding the final examination and registration of disabilities using a self-reporting method (64).

This study analyzed the time effects of health-related factors on the occurrence of new disabilities. As shown in Table 1, information on disabilities has been collected since 2010. Therefore, this study aimed to determine whether there is a new disability through registration. In other words, it was not known whether there was a pre-existing disability among the concerned population before the Korean Health Panel Survey, or whether the disability occurred during the survey period. Therefore, for this study, 359 people were under the category of Newly Occurred Disability from 2011 to 2018 (~8 years) and 7,013 were without disabilities from 2008 to 2018 (11 years), finally resulting in 7,372 participants for the study. Table 2 provides detailed information on the number of new individuals with disabilities that occurred during the study period (2008–2018).

**Table 2:** Newly occurred disability by years.

**Year**	**2008**	**2009**	**2010**	**2011**	**2012**	**2013**	**2014**	**2015**	**2016**	**2017**	**2018**	**Total**
Disability newly occurrence	0	0	Base line	*N =* 105	*N =* 20	*N =* 20	*N =* 25	*N =* 78	*N =* 34	*N =* 38	*N =* 39	*N =* 359
Non-disabled						*N =* 7,013						*N =* 7,372

Source: (2008–2018). The Korea Health Panel Survey (KHPS); N, Number of participants.

### 2.3. Dependent variable

The dependent variable in this study was the occurrence of new disabilities in participants (2011–2018). In the Korean Health Panel data, the presence or absence of new disabilities was reconstructed using the dichotomous method for newly occurring disabilities starting in 2010 and no disabilities from 2008 to 2018 (11 years).

### 2.4. Independent variables

This study aimed to investigate the time effect of health-related factors on newly developed disabilities. The covariates of health-related factors that can lead to new disabilities in this study were as follows: age, sex, education level, alcohol consumption, smoking, physical activity, stress and presence of disease, number of diseases, and types of chronic diseases.

The age of the participants ranged from 4 to 106 years. Age was analyzed by grouping the participants into eight groups as follows: 19 years or younger, 20 or more but <30, 30, or more but <40, 40 or more but <50, 50 or more but <60, 60 or more but <70, 70 or more but <80, and over 80 years of age. The level of education acquisition was first measured on a 9-point Likert scale. However, in this study, it was analyzed by restructuring into five groups (e.g., elementary school or lower, middle school, high school, university, junior college, and graduate school or higher). The income level was utilized in the fifth quintile of household income.

Chronic diseases were assessed using one or more duplicates for 21 choices of various chronic diseases, allowing participants to choose from the chronic diseases they suffered from in duplicate. This study was analyzed by restructuring the presence or absence of chronic diseases, the number of chronic diseases (e.g., 0, 1, 2, and more), and types of chronic diseases (e.g., high blood pressure, diabetes, hyperlipidemia, arthritis, tuberculosis, ischemic heart disease, and cerebrovascular disease). The definition of the prevalence of chronic diseases was presented in the Korean Health Panel Survey.

Smoking status was measured using two questionnaires: one to measure current/past smoking amount and the other, secondhand smoke level. First, the amount of current/past smoking was measured on a 4-point Likert scale, with the following questions: “Do you currently smoke?” which was to be answered by choosing any of the following options: (1) never smoked, (2) used to smoke but not now, (3) occasionally smoked, (4) and now smoking daily. Second, the questionnaire on second-hand smoke asked “How much time per day do you inhale other people's cigarette smoke indoors at work or at home?” which was to be answered by choosing any one of the following options: (1) 0 h, (2) <1 h, (3) more than 1 h to <2 h, and (4) more than 2 h.

Alcohol consumption was measured as a composite of three questions: whether or not drinking, the number of drinks per year, and the average amount of alcohol consumed on an; (a) 8-point Likert Scale Alcoholism “How often have you been drinking in the past year?” The questions were as follows: (1) Never drinking in life, (2) no alcohol in the past year, (3) less than once a month, (4) once a month, (5) 2–3 times a month, (6) once a week, (7) 2 weeks to three times, and (8) almost every day; (b) the number of drinks per year; (c) average alcohol consumption using a five-point Likert scale was calculated as “How many drinks do you usually drink on a drinking day?” and is measured as follows: (1) 1–2 glasses, (2) 3–4 glasses, (3) 5–6 glasses, (4) 7–9 glasses, and (5) 10 or more glasses.

Physical activity was assessed on an 8-point Likert scale with the following response options: (1) never, (2) 1 day, (3) 2 days, (4) 3 days, (5) 4 days, (6) 5 days, (7) 6 days, and (8) 7 days (daily). It was measured as a composite of three questionnaires: vigorous physical activity, moderate physical activity, and walking. The vigorous physical activity questionnaire used the question, “In the past week, on how many days did you do more than 10 min of strenuous physical activity that caused you to be out of breath and have a significant increase in heart rate?” The response options were: running, mountaineering, soccer, basketball, jumping rope, singles tennis, squash, and so on, swimming and biking fast only, including strenuous occupational activities such as carrying heavy objects, provided that walking is only very fast walking as applicable. The questionnaire on moderate physical activity asked the question: “In the past week, on how many days did you perform moderate-to-high physical activity for more than 10 min in which you had shortness of breath and a slight increase in heart rate?” The participants could choose their responses from options such as volleyball, badminton, table tennis, doubles tennis, yoga, gymnastics, and so on, including vocational activities such as carrying light objects, swimming, and biking slowly only, but walking a little faster). Finally, the questionnaire on walking comprised the question, “In the past week, on how many days did you walk for more than 10 min?” (This includes walking slowly or at a normal speed for commuting to work, other means of transportation, and walking fast for exercise).

Stress was assessed using a 5-point Likert scale with the following options for responses: (1) never, (2) occasionally, (3) often, (4) almost always, and (5) always. It was measured as a composite of mental and physical stress and task-dependent stress, where (a) Mental/physical stress was measured with “During the past month, have you ever felt that it was difficult to handle mentally and physically in your life?” and (b) task stress with “During the past month, have you had so much to do that you forgot to do something really important?”

### 2.5. Statistical analyses

Pearson's χ^2^ test and *t*-tests with counts and column percentages were performed for descriptive statistics. This study mainly analyzed the relationship between the occurrence of new disabilities and health-related factors and the number of chronic causes using parametric survival analysis with Weibull distribution (65). This study used the parametric model since the Cox proportional-hazards model makes minimal assumptions about the form of the reference risk function; however, this lack of assumptions hampers the prediction of risk and other related functions for a given covariate set. Therefore, the result estimation curve is not smooth and does not have information about what happens between observed times of failure. Contrarily, the parametric model provides a good and soft prediction assuming the functional form of risk, which is used by this study as the shape of the risk does not change significantly over time.

Furthermore, we preferentially performed additional analyses of the risk estimates from models that were adjusted for age, sex, income, education, smoking, alcohol consumption, physical activity, and stress. Eight additional survival analyses were employed to determine the relationship between the presence of chronic disease and the occurrence of new disabilities according to the type of chronic disease. The specific additional factors are as follows.

Additional Model 1: Main survival analysis of health-related factors plus dichotomous chronic disease.
Additional Model 2: Main survival analysis of health-related factors plus the type of chronic disease (hypertension).
Additional Model 3: Main survival analysis of health-related factors plus the type of chronic disease (diabetes).
Additional Model 4: Main survival analysis of health-related factors plus the type of chronic disease (hyperlipidemia).
Additional Model 5: Main survival analysis of health-related factors plus the type of chronic disease (arthropathy).
Additional Model 6: Main survival analysis of health-related factors plus the type of chronic disease (tuberculosis).
Additional Model 7: Main survival analysis of health-related factors plus the type of chronic disease (ischemic heart disease).
Additional Model 8: Main survival analysis of health-related factors plus the type of chronic disease (cerebrovascular disease).

All statistical analyses were performed at a significance level of ≤ 0.05, with a confidence interval of 95%, using StataCorp Stata 15.1.

## 3. Results

This study was a survival analysis of the relationship between the occurrence of new disabilities and health-related factors and chronic diseases. Descriptive statistics of the study population are shown in Table 3. During the study period (2008–2018), a total of 7,372 people participated, and it was noted that 359 new disabilities occurred from 2011 to 2018; the years 2008 and 2009 had no record of disability. As of 2018, the average age was 52.14 years (SD = 21.39 years) with 3,503 (47.52%) men. It was found that 50% of the participants had chronic diseases, whereas ~21% (1,539) and ~16% (1,174) had two and more diseases, respectively. A total of 1,041 of three or more chronic diseases were identified. The most common chronic diseases among participants were hypertension (2,284), followed by arthropathy, hyperlipidemia (1,483), diabetes (1,006), cardiovascular disease (365), cerebrovascular disease (300), and tuberculosis (55).

**Table 3:** Descriptive statistics descriptive statistics of study sample with disability (year of 2018).

		**% (** ***N*** **), Mean (SD)**		
**Variables**	**Disability**	**Non-disability**	**Overall**	***p*** **-value**
	***N** =* **359 (4.87%)**	***N** =* **7,013 (95.13%)**	***N*** =**7,372 (100%)**	
Age (continuous)	65.13 (SD = 18.48)	51.47 (SD = 21.32)	52.14 (SD = 21.39)	<0.01
**Age (categories)**				<0.01
19 ≤	4.74% (17)	8.68% (609)	8.49% (626)	
20 ≤ -30 <	1.67% (6)	13.69% (960)	13.10% (966)	
30 ≤ -40 <	2.79% (10)	7.67% (538)	7.43% (548)	
40 ≤ -50 <	7.24% (26)	13.63% (956)	13.32% (982)	
50 ≤ -60 <	10.31% (37)	15.69% (1,100)	15.42% (1,137)	
60 ≤ -70 <	20.33% (73)	15.97% (1,120)	16.18% (1,193)	
70 ≤ -80 <	36.21% (130)	16.11% (1,130)	17.09% (1,260)	
80 ≤	16.71% (60)	8.56% (600)	8.95% (660)	
Male	51.81% (186)	47.30% (3,317)	47.52% (3,503)	0.10
**Education**				<0.01
≤ Elementary	11.70% (42)	3.14% (239)	3.81% (281)	
≤ Middle school	52.65% (189)	28.42% (1,993)	29.60% (2,182)	
≤ High school	24.79% (89)	30.20 % (2,118)	29.94% (2,207)	
≤ Colleges	10.31% (37)	29.06% (2,038)	28.15% (2,075)	
> Graduate school	0.56% (2)	8.91% (612)	8.51% (627)	
**Income 5th quintile**				<0.01
1st	50.14% (180)	23.04% (1,616)	24.37% (1,796)	
2nd	20.61% (74)	19.69% (1,381)	19.74% (1,455)	
3rd	13.37% (48)	18.27% (1,281)	18.03% (1,329)	
4th	7.80% (28)	19.45% (1,364)	18.88% (1,392)	
5th	7.80% (28)	19.55% (1,371)	18.98% (1,399)	
Smoking (Min *=* 0, Max = 3)	0.37 (SD = 0.75)	0.28 (SD = 0.69)	0.29 (SD = 0.69)	<0.05
Drinking alcohol (Min *=* 0, Max = 11)	2.20 (SD = 3.24)	3.21 (SD = 3.46)	3.16 (SD = 3.45)	<0.01
Physical activity (Min *=* 0, Max = 21)	4.79 (SD = 4.40)	5.55 (SD = 4.88)	5.52 (SD = 4.86)	<0.01
Stress (Min *=* 0, Max = 8)	1.05 (SD = 1.63)	0.92 (SD = 1.28)	0.92 (SD = 1.30)	0.13
Chronic disease (Yes)	84.40% (303)	49.21% (3,451)	50.92% (3,754)	<0.01
**Chronic diseases**				<0.01
0	15.60% (56)	50.79% (3,562)	49.08% (3,618)	
1	22.84% (82)	20.78% (1,457)	20.88% (1,539)	
2	25.35% (91)	15.44% (1,083)	15.93% (1,174)	
≥ 3	36.21% (130)	12.99% (911)	14.12% (1,041)	
**Major types of chronic diseases**				
Hypertension	76.69% (243)	29.10% (2,041)	30.98% (2,284)	<0.01
Diabetes	44.01% (158)	12.09% (848)	13.65% (1,006)	<0.01
Hyperlipidemia	36.21% (130)	19.29% (1,353)	20.12% (1,483)	<0.01
Arthropathy	39.28% (141)	27.26% (1, 912)	27.85% (2, 053)	<0.01
Tuberculosis	10.31% (37)	0.26% (18)	0.75% (55)	<0.01
Ischemic heart disease	11.98% (43)	4.59% (322)	4.95% (365)	<0.01
Cerebrovascular disease	11.98% (43)	3.66% (257)	4.07% (300)	<0.01

N = 7, 372; Source: 2008–2018 the Korea Health Panel Survey (KHPS); N, number of participants; SD, Standard deviance; Min, Minimum; Max, Maximum.

### 3.1. Result of the main analysis

As shown in Table 4, the age of the participants were not statistically significant in developing new disabilities at all in follow-up visits, whereas sex was statistically significant (HR = 0.45, 95% CI = 0.35–0.57). The hazard of developing new disabilities was lower for female participants than for male participants. Higher education attainments were associated with lower hazard of developing new disabilities; HR estimates decreased from 1 to 0.54 to 0.38 to 0.23 to 0.06, relative to the referent group, below middle school (HR = 0.54, 95% CI = 0.38–0.77), below high school (HR = 0.38, 95% CI = 0.24–0.58), below colleges (HR = 0.23, 95% CI = 0.14–0.40), and greater graduate school (HR = 0.23, 95% CI = 0.01–0.25). It was found that the higher the income levels in the fifth quintile, the lower the risk ratio for new disability at all times of follow-up compared to the lowest level of income, which is the second level of income (HR = 0.73, 95% CI = 0.55–0.98, third level of income: HR = 0.71, 95% CI = 0.50–1.03, fourth level of income: HR = 0.47, 95% CI = 0.30–0.73, and fifth level of income: HR = 0.53, 95% CI = 0.33–0.84).

**Table 4:** Parametric survival (Weibull) regression of newly developed disability.

**Covariates**	**HR**	**95% CI**
**Age (in years)**		
19 ≤	-	-
20 ≤ -30 <	0.63	(0.24, 1.68)
30 ≤ -40 <	1.25	(0.54, 2.90)
40 ≤ -50 <	1.23	(0.61, 2.49)
50 ≤ -60 <	0.81	(0.39, 1.68)
60 ≤ -70 <	0.74	(0.36, 1.52)
70 ≤ -80 <	0.74	(0.36, 1.53)
80 ≤	0.49	(0.23, 1.03)
Female	0.45^***^	(0.35, 0.57)
**Education**		
≤ Elementary	-	-
≤ Middle school	0.54^**^	(0.38, 0.77)
≤ High school	0.38^***^	(0.24, 0.58)
≤ Colleges	0.23^***^	(0.14, 0.40)
> Graduate school	0.06^***^	(0.01, 0.25)
**Income quintile**		
1st	-	-
2nd	0.73^*^	(0.55, 0.98)
3rd	0.71	(0.50, 1.03)
4th	0.47^***^	(0.30, 0.73)
5th	0.53^**^	(0.33, 0.84)
Smoking	1.25^**^	(1.07, 1.46)
Alcohol consumption	0.91^***^	(0.88, 0.95)
Physical activity	0.97^*^	(0.94, 0.99)
Stress	1.06	(0.98, 1.15)
**Number of chronic diseases**		
0	-	-
1	2.75^***^	(1.70, 4.44)
2	3.66^***^	(2, 22, 6.04)
≥ 3	5.45^***^	(3.30, 9.00)

* P < 0.05,

** P < 0.01,

*** P < 0.001; 2008–2018 the Korea Health Panel Survey (KHPS); HR, hazard ratio; 95% CI, confidence interval.

**Figure 1:**
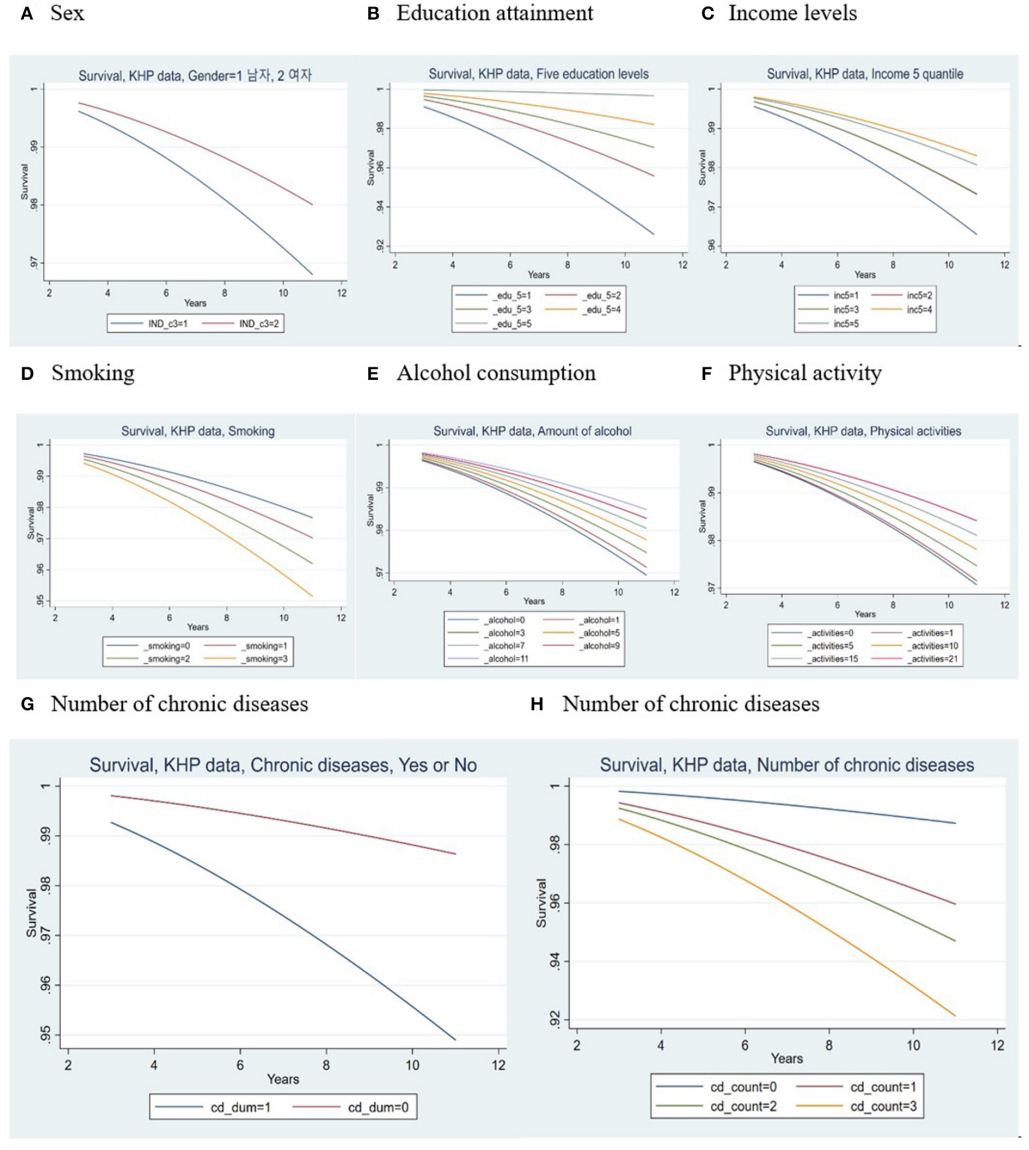
PGraph representing parametric survival (Weibull) regression of newly developed disabilities. **(A)** Sex; **(B)** Education attainment; **(C)** Income levels; **(D)** Smoking; **(E)** Alcohol consumption; **(F)** Physical activity; **(G, H)** Number of chronic diseases.

In this study, the health factors that could be related to the occurrence of new disabilities were smoking, alcohol consumption, physical activity, and stress (Figure 1). An increase in each unit in smoking was significantly associated with an increased risk of newly developing disability at all follow-up visits (HR = 1.25, 95% CI = 1.07–1.46). Similarly, an increase in each unit of alcohol consumption was linked to an increased risk of newly developing disability at all follow-up visits (HR = 0.91, 95% CI = 0.88–0.95), which was highly statistically significant (*p* < 0.0001). However, with a unit's increase in physical activity, the risk of developing a disability was significantly reduced at all follow-ups (HR = 0.97, 95% CI = 0.94–0.99).

As seen in the Figure 1, higher scores on the number of chronic diseases (valid scores = 0, 1, 2, 3, or more) represented a greater level of newly developing disability present at all follow-ups. This shows that higher (“worse”) values of chronic diseases were associated with poorer prognosis of disability development (Hazard ratio estimates increase from 1 to 2.75 (95% CI = 1.70–4.44) to 3.66 (95% CI = 2.22–6.04) to 5.45 (95% CI = 3.30–9.00), relative to the referent group of the number of chronic diseases = 0). This difference was highly statistically significant (*p* < 0.0001).

**Figure 2:**
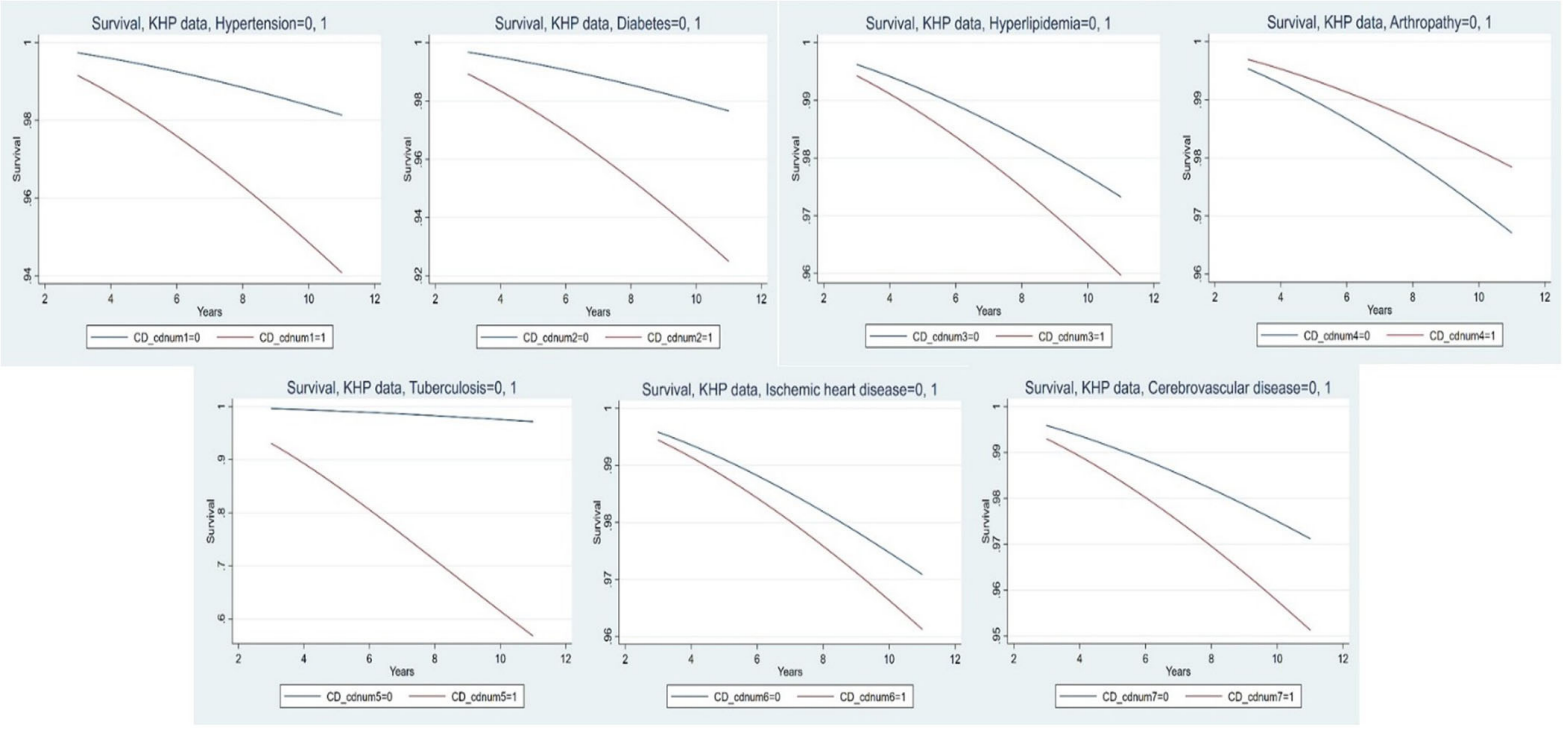
Major chronic diseases of newly developed disability.

Table 5 and Figure 2 provide more specific information on chronic diseases related to newly developed disabilities during the study period. The participants with chronic diseases had an ~4-fold increased risk of developing a new disability at all follow-ups compared to those without chronic diseases (HR = 3.81, 95% CI = 2.47–5.88). When looking at the increase in the risk of developing new disabilities due to chronic diseases, participants with tuberculosis showed the highest risk (HR = 19.87, 95% CI = 13.93–28.36), followed by diabetes (HR = 3.29, 95% CI = 2.63–4.12), hypertension (HR = 3.25, 95% CI = 2.45–4.30), cerebrovascular disease (HR = 1.71, 95% CI = 1.23–2.37), and hyperlipidemia (HR = 1.52, 95% CI = 1.21–1.91). An interesting study result was that participants with arthropathy showed a statistically significant decrease in the risk of newly occurring disability at all follow-up periods (HR = 0.65, 95% CI = 0.51–0.84).

**Table 5:** Additional analyses of parametric survival (Weibull) regression of newly developed disability.

**Chronic diseases (Yes or No)**	**HR**	**95% CI**
Model 1:	3.81^***^	(2.47, 5.88)
**Major types of chronic diseases**		
Model 2: Hypertension	3.25^***^	(2.45, 4.30)
Model 3: Diabetes	3.29^***^	(2.63, 4.12)
Model 4: Hyperlipidemia	1.52^***^	(1.21, 1.91)
Model 5: Arthropathy	0.65^**^	(0.51, 0.84)
Model 6: Tuberculosis	19.87^***^	(13.93, 28.36)
Model 7: Ischemic heart disease	1.34	(0.96, 1.86)
Model 8: Cerebrovascular disease	1.71^**^	(1.23, 2.37)

^*^P < 0.05,

** P < 0.01,

*** P < 0.001; 2008–2018 the Korea Health Panel Survey (KHPS); HR, hazard ratio; 95% CI, confidence interval.

## 4. Discussion

This longitudinal study confirmed the relationship between health-related factors and specific chronic diseases. Its findings can be used as a crucial foundation for establishing healthcare policies and services that can lower and prevent disability by preventing and reducing specific negative health behaviors and unhealthy behavioral factors, and alleviating chronic diseases.

Among socioeconomic factors, the relationships between sex, levels of educational attainment, income, and the occurrence of new disabilities were confirmed. Sex was statistically significant with predicting disability and death as in other study (19). As expected from previous studies, this study identified the fact that the higher the levels of education and income, the lower the risk of disability compared to the lower the education level, which was in line with the existing research (8, 9, 14, 17, 18, 20). A study of heterogeneous trajectories and associated risk factors in the development of sensory-cognitive coupling measures based on the common-cause hypothesis found that several easily identifiable socioeconomic and health-related risk factors, such as low financial status of a household, were subsequently identified as sensory-cognitive risk factors, which are reported to be an initial indicator of reduction (8). A study of the high prevalence of disability and HIV risk among urban adults with low socioeconomic status in 17 cities in the United States (14) found that adults with low socioeconomic status and heterosexual activity reported a higher prevalence of disability and differences in health, healthcare access, and risk factors. Studies investigating the prevalence and related factors of frailty and disability among older adults in China, Ghana, India, Mexico, Russia, and South Africa found that higher education and income levels were associated with lower levels of frailty and disability (17). It is believed that those above a certain level of income and a certain level of education may have more opportunities to focus more on health-related personal hygiene and health behaviors and healthcare that can reduce and eradicate chronic diseases.

Various studies have been conducted on health behaviors and choices as the causes of disability [e.g., physical activity (19), consuming fruits and vegetables less than once a day (32), current smoking/short-term ex-smoking (32), never/former/heavy alcohol drinking (32)]. A cross-sectional study of Singapore's older adult population found that a combination of factors, such as cognitive deficiency, old age, women, Malay and Indian ethnicity, lack of education, retirement or homemaker status, and the presence of chronic diseases (especially stroke, heart problems, depression, and dementia) were significant. A longitudinal study using modifiable midlife risk factors to predict the risk of physical disability during old age was also investigated, which confirmed the relationship between potentially modifiable risk factors in middle age and disability after 13 years of age (19). A study found that disability is associated with smoking, obesity, the female sex, and diabetes in predicting disability and death in the Australian population (19). A nationwide cross-national study that explored factors related to reduced mobility leading to disability in adults aged 20–89 years, found that an increase in the age of participants over 40 years was associated with a new decrease in mobility, and lifestyle habits of participants younger than 40 years, including reduced physical activity in women and being overweight, which were in turn, associated with reduced mobility at all levels (27).

In a three-city Dijon cohort study (32) it was found that unhealthy behaviors and disabilities in older adults were examined for individual and combined associations of unhealthy lifestyle behaviors and disability risk in France. Low/moderate levels of physical activity, consumption of fruits and vegetables less than once per day, and currently smoking/short-term smoking were independently associated with an increased risk of disability, whereas no strong association was found with alcohol consumption. In that study, it was found that the risk of disability increased progressively with the number of unhealthy behaviors, which were independently associated with disability (32). Participants who engaged in all three unhealthy behaviors (e.g., less physical activity, less consuming fruit and vegetables, current smoking/short term ex-smoking) had a significantly increased risk of disability compared to those who did not. The association between unhealthy behavioral scores and disability was explained by body mass index (BMI), cognitive function, depressive symptoms, trauma, chronic conditions, cardiovascular disease, and its risk factors. An unhealthy lifestyle is associated with a greater risk of thinking disorders, which risk progressively increases with the number of unhealthy behaviors (32). Chronic conditions, depressive symptoms, trauma, and BMI partially explained this association (32). Although factors related to fruit and vegetable intake could not be analyzed in the current study, their regular intake was found to delay the onset of the disability and maintain physical health. Furthermore, drinking was not found to be a statistically significant factor in the occurrence of new disorders in this study (32). This means that the variation according to the amount and frequency of drinking among the participants in this study was too large, and most did not drink frequently. In this study, a number interpreted as a factor in the occurrence of disability was obtained, which was, however, not statistically significant.

In this study, depression and anxiety were also analyzed as factors in the development of disabilities. To investigate the relationship between baseline depressive symptoms and the development of dysfunction (ADLs: activities of daily living, IADLs: instrumental activities of daily living, mobility) at the 2-year follow-up, a prospective cohort study investigated the onset of depressive symptoms and dysfunction for 2 years. It was confirmed that the onset of IADL disorder and the onset of mobility impairment were consistently higher in the Chinese-American elderly with high levels of depression (66). Studies have identified the effects of anxiety and/or depressive disorder (ADD) and chronic somatic disorder (CSD) on disability and work disability, which are associated with significant levels of health-related disorders and work-related disorders (47). Both CSD and ADD cause significant disabilities, absenteeism, and attendance among the general population; however, the impact of ADD far exceeds that of CSD. A prior study found that CSD and ADD have synergistic effects on disability, supporting the current view that patients with physical and mental comorbidities form a severe subgroup with adverse prognoses (47).

Studies on the role of general mental and physical disorders in partial physical disorders worldwide have estimated the relationship between individuals (i.e., outcomes for individuals with disabilities), social influences (i.e., partial disorders avoided in society owing to disabilities), and mental and physical disabilities (52). Mental disorders (especially post-traumatic stress disorder, depression, and bipolar disorder) have more days of disability than physical disability. Mental and physical disabilities significantly impact partial disabilities at both individual and social levels. It was found that physical disability had a greater effect on partial disability than on mental disability (52). It should be recognized that the occurrence of disorders, such as anxiety and depression, plays a significant role not only in physical health behaviors and chronic diseases but also in psychological aspects. Therefore, individual and social efforts are needed to manage stress and relieve depression and anxiety.

Many studies have investigated the relationship between obesity and BMI levels and chronic diseases [e.g., hypertension (22), diabetes (36)], on possible causes of disability. A study investigating the joint association of obesity and hypertension and the risk of disability in a cross-sectional setting with community-dwelling older people in Shanghai (22) showed that the risk of ADL disorder is progressively greater in obese people with high blood pressure and IADL disorders greater in underweight individuals. Wong and colleges estimated the effects of past and future changes in obesity and diabetes prevalence in middle-aged individuals on the disability prevalence in adult Australians (36). If the percentage of obesity and diabetes observed in 2000 was as low as that observed in 1980, it was estimated that the prevalence of disabilities between the ages of 55 and 74 would have been low in 2010 (36). However, if the prevalence of obesity and diabetes were as high as the figure expected in 2025, the prevalence of disabilities would increase (36).

A prior study also investigated the association between type 2 diabetes and disability. It found that diabetes significantly increases the risk of disability in a study on the contribution of diabetes risk factors and complications (45). Obesity and a history of cardiovascular disease accounted for the largest proportion of the relationship between diabetes and disability, suggesting that weight and cardiovascular disease management can help control diabetes-related disorders (45).

A study investigating the association between obesity and disability development during adolescence to evaluate the longitudinal relationship between the two (26) found that the occurrence of obesity and persistent obesity during adolescence was significantly associated with an increased disability in young adulthood. In a Swedish population-based cohort study (29) that explored a potential bidirectional association between mobility impairment and obesity, it was found that obesity at baseline was associated with incident mobility disability. Bi-directional and longitudinal associations between weight and incident mobility disability were identified, along with an increased risk of developing the combination of two over time. As in other studies, diabetes and hypertension were found to significantly affect the occurrence of disabilities as also found in this study. However, while obesity or high BMI levels have been shown to affect disability in other studies, in this study, all participants with obesity or high BMI levels were normal (without any disability). Therefore, it was not possible to analyze the effects of obesity or a high BMI on the occurrence of new disorders.

A study on the relationship between chronic medical conditions caused by lack of physical activity and functional limitations over time investigated whether long-term physical activity could prevent functional limitations in the context of chronic disease accumulation among middle-aged and older adult populations (39). Faster development of chronic diseases and a steeper decline in activity are associated with a greater increase in functional limitations over time. Among those with faster-than-average chronic disease progression, those who remained active had a slower progression of functional restriction than those whose activity decreased more rapidly. This study, reflecting the findings of a previous study, provides evidence of an age-dependent buffering effect of activity maintenance on the longitudinal relationship between chronic conditions and functional limitations (39). Schultz-Larsen et al. (28) found that in a 14-year follow-up study of how physical activity (PA) for non-disabled older adult women and men relates to disability processes, the basic processes and effectiveness of disability development persisted permanently after adjusting for age, baseline vulnerability, and disability ratings, and low PA was independently associated with risk of accidents in males.

Leisure activity (LA) and disability trajectories of those over 11 years in Taiwan confirmed the LA trajectory and late disability of the concerned population (31). Participants in the consistently high or increasing LA trajectory group were more likely to be functionally independent, but participants in the decreasing LA subgroup had a higher risk of developing the disorder (24). The findings suggest that long-term changes in LA over time are beneficial to the physical health of older adults. A study of the osteoarthritis initiative evidence on disability and physical activity in the older population evaluated the relationship between changes in physical activity and disability in early inactive adults with or at high risk of knee osteoarthritis (34). It found that increased physical activity showed a differential relationship with improved disability scores in the late-life disability instrument limit and frequency scores (34). Increasing moderate-vigorous activity adhering to guideline levels showed the greatest decrease in developing disabilities, whereas an insufficient increase in physical activity was associated with increased disability (34).

A study on the relationship between chronic medical conditions caused by physical activity and functional limitations over time investigated whether long-term physical activity could prevent functional limitations in the context of chronic disease accumulation among middle-aged and elderly people (38). A faster accumulation of chronic disease and a steeper decline in activities are associated with a greater increase in functional limitations over time. Among those who increased their status faster than the average, those who remained active had a slower progression of functional limitation than those whose activity decreased more rapidly. This study provides evidence of an age-dependent buffering effect of activity maintenance on the longitudinal relationship between chronic conditions and functional limitations (38). Men benefit more from midlife leisure-time physical activity than women with the development of later disability, as found by the CORA Age study. As per a study, increasing physical activity reduces the risk of becoming disabled and delays the onset of disability by several years; however, it cannot be identified as influencing the severity of the disability (33, 42). Moreover, it found that men appear to benefit more from physical activity in their spare time than women (42). Various appropriate physical activities not only maintain and promote daily health but also directly or indirectly affect the occurrence of disability.

A study on the contribution of chronic disease to the burden of disability across smoking categories in middle-aged Belgian adults assessed the contribution of chronic disease to the burden of disability across smoking categories (48). The study also found an increasing trend in disability prevalence across both sexes in the smoking category. Furthermore, it found that musculoskeletal conditions have significantly contributed to the disability burden in men and women across all smoking categories (48). Other significant contributors to disabilities were depression and cardiovascular disease among non-smokers; depression, chronic respiratory disease, and diabetes in former smokers; chronic respiratory disease, cancer, and cardiovascular disease in daily light smokers; cardiovascular and chronic respiratory diseases in men; and depression and diabetes in women who smoked every day (48). In addition to the wellknown effects of smoking on mortality, our findings showed an increasing trend in disability prevalence and various contributors to the disability burden across smoking categories (48). This information could be useful in defining Belgium's disability reduction strategy from a public health perspective.

A study on the association between smoking and early retirement due to chronic disability showed the long-term effects of smoking on disability retirement in Sweden (49). Smokers are more likely to receive a (full) disability pension due to the quick onset of disabilities in them. However, in models with the sibling fixation effect, the size of the effect was reduced by more than one-third (49). The results thus highlight the importance of confounding factors, such as childhood situations or behaviors, which have not been accounted for in previous studies. It also considers the impact of disabilities on various health conditions. In relative terms, the impact is greatest for circulatory conditions and tumors (49). Although it could not be confirmed in this study, drug abuse along with smoking has a significant effect on the occurrence of disability, and illicit drugs and prescription medications can all be substances of abuse. People with disabilities may have multiple risk factors that can increase their risk of substance abuse. Smoking and drug misuse did not help maintain health and were confirmed again in this study as important factors that eventually cause disability (67).

This study also confirmed that chronic diseases were closely related to the occurrence of new disabilities. A study evaluating the incidence and determinants of physical disability in a modern and nationally representative sample of U.S. military veterans confirmed that old age, marriage/cohabitation, and various health conditions, especially diabetes, “cardiovascular diseases,” and chronic pain, are associated with an increased risk of physical disability and IADL (e.g., food and drug compliance) disability (35). A study of disability status as a precondition for chronic disease found that adults with chronic physical disabilities were more likely to have chronic diseases such as coronary heart disease, cancer, diabetes, and high blood pressure than adults without any such conditions (41). A subgroup of people with lifelong disabilities (i.e., physical, mental, intellectual/developmental, and sensory) experienced a similarly increased probability of chronic disease compared to those without restrictions. Adults with a lifetime disability were more likely to have chronic diseases than those without limitations, indicating that having a disability is likely to increase the risk of poor health (41). This distinction is important for understanding how to prevent health risks in people with disabilities. There is a need for health promotion efforts targeting people with disabilities. In a prospective cohort study, in a large cohort of older Australian women, the onset and progression of chronic diseases and disorders showed that those who reported the presence of chronic disease were more likely to experience the disability than those who died without any disability (68).

In the study on the incidence of disability and functional decline in older adults with major chronic diseases, the pattern of functional loss was investigated, and the onset and sequence of ADL disorders were compared with those without these diseases (69). Older adults with major chronic conditions were found to have a higher incidence of disability in all ADL items. People with the disorder developed chronic diseases earlier than those in the control group. The order of ADL loss, sorted by the median age of onset of disability among people with major chronic disease, is bathing, walking, getting dressed, having a bowel movement, moving, and eating (69). The sequence of losses derived for the control group was largely similar, but disability progression for patients with major chronic disease was compressed within a shorter period, and the timing gaps between adjacent disorders were smaller (69). Older adults with major non-communicable diseases face a faster and steeper decline gradient (69). Chronic care delivery programs thus must adapt to the dynamic changes in the functional status of older patients. Health interventions that help patients delay the onset of disability and optimize functional autonomy within new chronic treatment models should specifically target early loss of activities (e.g., bathing, dressing, and walking). This can help individuals avoid disabilities and retain their agility for a longer period of time in their life. The study of the effects of type 2 diabetes and high depression symptoms on the development of daily life (ADL) disability and mortality potential in older Puerto Ricans investigated the development of ADL disability and mortality activities in accordance with diabetes and high depression symptoms among adults over 60 years of age and confirmed that diabetes and high depression symptoms are the risk factors for ADL disability and mortality in older Puerto Ricans (40).

Nusselder et al. investigated the contribution of chronic diseases to disability in French men and women (46). It found that musculoskeletal disorders have caused most disabilities in both men and women, followed by cardiovascular disease in men, and in women closely followed by anxiety-depression (especially in women) (46). The high rate of incidence of musculoskeletal disorders in women reflects their higher prevalence and impact on disability. Moreover, it was noted that excluding the institutionalized population did not change the overall conclusions. The number one cause of the higher prevalence of disability in women than men is the high prevalence of moderately disabled conditions (46). While traditional disabling conditions, such as musculoskeletal disorders, are more prevalent and disabling among women, fatal diseases, such as cardiovascular disease, are also significant contributors to women and men (46). The Health and Retirement Study on Chronic Obstructive Pulmonary Disease (COPD) and Cognitive Impairment and Disability Development investigated the prevalence and cumulative incidence of disability in adults with and without COPD and the association of COPD with cognitive impairment and disability (50). Both COPD and mild cognitive impairment increase the risk for disability (50). The risks imposed by COPD are significant and comparable to or higher than those of other chronic diseases (e.g., stroke and diabetes) (50).

A systematic review of the prevalence of disability in people with cancer, cardiovascular disease (CVD), chronic respiratory disease, and/or diabetes investigated the prevalence of disabilities associated with cancer, CVD, chronic respiratory disease, and diabetes was investigated (57). The prevalence of difficulties in activities of daily living (e.g., eating, bathing, and dressing) has been reported extensively in cancer survivors, CVD patients, chronic respiratory tract patients, and diabetic patients (57). Many people with the above-mentioned conditions experience some form of disability (57). The dysfunctional trajectory estimate projected an association between diabetes, heart disease, and dementia, which may increase self-management difficulty in those afflicted by it (51). People with the potential for dementia, and diabetes, or those with diabetes and heart disease were significantly more likely to have a mild disability trajectory than those without such disability (51). People with potential for dementia were significantly more likely to have a severe disability trajectory than those with a non-disabled trajectory (51). Several chronic and cognitive conditions can be useful for health policymakers to make decisions about treatment provision and services. This study confirmed that the management of chronic diseases is very important for suppressing and delaying the occurrence of disorders.

Furthermore, it was confirmed that people with a greater number of chronic diseases were more exposed to environments that could cause disability. Multiple chronic diseases (MCC) on activity limitation in Mexican-American older care recipients and the effect of MCC on basic ADL or IADL limitations were investigated in a prior study (30). The MCCs selected for the analysis were diabetes, hypertension, stroke, heart disease, arthritis, emphysema/COPD, cognitive impairment, depression, and cancer. Managed participants with three or more chronic conditions were more likely to have mobility, self-management, ADL, and IADL restrictions. Among the managed participants, those with arthritis, hypertension, and cognitive impairment were significantly more likely to have limited mobility than those with arthritis or hypertension alone (30). The MCC was more strongly associated with ADL and IADL restriction among management groups, especially for those with hypertension and arthritis, diabetes, cognitive impairment, or heart disease (30). A study of the association of chronic disease patterns with visual impairment and healthcare use found that individuals in the four multimorbidity groups were at a higher risk of visual impairment compared to the healthy group (43). The characteristics of the high-risk groups identified in this study may aid in the development and implementation of interventions to avoid the serious consequences of complex chronic diseases (43).

Sheridan et al. used a hierarchical model to compare respondents with multiple chronic diseases to healthy respondents, using a hierarchical model to compare unique multimorbidity combinations, future disability, and poor self-assessment health (SRH) in older adult Europeans were investigated (70). Multimorbidity combinations with high depressive symptoms were associated with an increased probability of reporting poor SRH and an increased rate of ADL-IADL disorders (70). It was more likely to be more disabling than the combination containing only the physical condition (70). These findings argue for the continued integration of mental and physical chronic diseases in the conceptualization of multimorbidity and have important implications for clinical practice and healthcare delivery.

However, in this study, the results of predicting a negative relationship with the possibility of new disorders for hip joint disease were obtained (71). Previous studies of the hip joint have been shown to make a significant contribution to the development of disability (71). Problems with the hip joint are known to cause a number of physical pain and disability. Therefore, in Korea, it is very well-recognized that the disease of the hip joint causes poor quality of life and disability throughout the body. Therefore, when Koreans suffer from hip joint disease, it seems that rapid diagnosis and correct joint management over a long period of time do not develop into a serious disease that can cause disability. Moreover, Exercise or physical activity is related to the occurrence of disability. Arthrosis causes a lack of physical activity, and this lack of physical activity is the cause of new disability in the long term (72). Due to the proportion of arthropathy patients is higher among disability developed group compared to non-developed groups in our study, it could possibly associate with controlling physical activity which would be possible pathway of developing disability in multivariate model.

The Survey on the Status of Persons with Disabilities 2020 in South Korea (3) showed ~80% of disabled individuals over 19 years of age have chronic diseases, with an average of 2.2 chronic diseases per disabled person. The chronic diseases include high blood pressure, back and neck pain, osteoarthritis, and diabetes mellitus. In general, the physical and mental quality of life of individuals with disabilities is lower than that of the general population (73). Among the disabled, those who thought their health status was “good,” accounted for 14.0%, which is less than half of the total population (32.4%) in Korea, and the rate of experiencing depression and stress in life was high (73). A total of 32.1% of people with disabilities needed support from others in their daily lives, which was slightly lower than the 33.9% reported in 2017 (73). Among them, the number of cases where “almost everything requires the support of others” was 6.2%, an increase from 2017 (5.5%) (73).

The quality of life of disabled people is significantly lower than that of non-disabled people all across the world (2, 67, 73). According to the US CDC's Report on the Related Health Status of People with Disabilities (67), people with disabilities have poor overall health, poor access to adequate healthcare, high rates of smoking, and lack of physical activity. People with disabilities need healthcare and wellness programs for the same reasons as everyone else. Being healthy essentially means staying healthy, so we can lead a full and active life. This means having the tools and information to make healthy choices and knowing how to prevent the disease. For people with disabilities, this also means knowing that the health problems associated with disability can be treated.

This study confirmed that health-related factors (e.g., drinking, smoking, physical activity, and chronic diseases) are significantly associated with the development of new. In Korea, the health care sector is rapidly changing due to the steady increase in income level, aging population, increase in chronic diseases, strengthening of medical insurance coverage, various treatment practices and medical technology development, etc., and the increase in medical expenses is expected to further accelerate. Accordingly, in order to prepare the basis for improving the policy goals of efficiency, effectiveness, and equity in the health and medical sector, and to optimize the burden on health finance, the health level and health behavior basic data on medical use and medical expenses are first used. can be used This study identified the dynamic structure of the individual's health level, which is the cause of medical expenses, in various and complex ways in the mutual process of personal factors and social, physical, and environmental factors. It is expected that scientific basic data of health care policy will be prepared through multidisciplinary analysis and research of these series of processes and will be used as basic data for health care policy and health insurance policy establishment.

This study had the following limitations. First, this study is a longitudinal analysis of socioeconomic factors, health behaviors and choices, and chronic diseases that can factor in the occurrence of new disabilities. Nevertheless, it should be noted that it is difficult to interpret the results of this study as a causal relationship between the occurrence of new disorders. Second, this study utilized a multiple-item and self-reported measurement to assess all study variables from the participants' 1-day recollection. Therefore, this study's results may be inaccurate or biased. Furthermore, people may overestimate their health levels and behaviors due to societal bias. However, this study strengthens the literature on disability-induced factors. Factors that can cause disability can be influenced by the reciprocal processes of social, physical, and environmental factors. Third, the limited sampling region warns against the generalization of research findings to those living in more diversely populated areas (e.g., Whites, Blacks, and Hispanics). Third, as this study was conducted in the Republic of Korea, its results cannot be generalized to other countries.

## 5. Conclusion

In Korea, the incidence of new disabilities is gradually increasing owing to an aging population and an increase in chronic diseases. This study analyzed panel data that could be investigated in depth, including the factors affecting the occurrence of new disorders. An individual's health status and level of fitness, which is the cause of the occurrence of new disabilities, is variously and intricately intertwined in the mutual process of personal factors and social, physical, and environmental factors and has a dynamic circulation structure. Through multidisciplinary analysis and research on these processes are needed, this study can be used to prepare basic scientific data for health and medical policies to suppress and delay the occurrence of new disorders or disabilities. Accordingly, to alleviate the social and economic burden caused by the occurrence of disability, the first priority is to use medical care and spend on medical expenses in order to provide the basis for improving the policy goals of efficiency, effectiveness, and equity in the healthcare sector, and to optimize the burden on health finance. Basic data are further needed, and for establishing various healthcare and health insurance policies, but also to greatly contribute to the promotion of related academic research fields. It will aid in preventing health problems related to the occurrence of disability much in advance; maintaining and protecting health; supplementing and correcting the socioeconomic imbalance of health factors that cause disability; and reducing population, social, economic, and health-related factors. Thus, largely, it can be used as background information for the development of medical services and systems that can reduce and prevent the occurrence of disabilities in Koreans in the future.

To protect one's health the following basic steps should be followed to improve lifestyle and eliminate and reduce risk factors for disability (67): getting regular exercise, eating a healthy diet and losing weight if necessary, reducing stress, and making lifestyle changes, such as getting enough sleep. Following the above-mentioned steps can significantly lower the risk of developing chronic diseases that can lead to disorders such as heart disease, diabetes, and cancer. Thus, it can be said that maintaining an active and healthy lifestyle can considerably help avoid disabilities in the general population, which can further help minimize the risk of socioeconomic burden.

## Data availability statement

The original contributions presented in the study are included in the article/Supplementary material, further inquiries can be directed to the corresponding author/s.

## Author contributions

TK and I-HO wrote the paper. All authors participated in the research concept of the paper and have read and agreed to the published version of the manuscript.
